# The Artificial Kidney Initiation in Kidney Injury 2 (AKIKI2): study protocol for a randomized controlled trial

**DOI:** 10.1186/s13063-019-3774-9

**Published:** 2019-12-16

**Authors:** Stéphane Gaudry, David Hajage, Laurent Martin-Lefevre, Guillaume Louis, Sébastien Moschietto, Dimitri Titeca-Beauport, Béatrice La Combe, Bertrand Pons, Nicolas de Prost, Sébastien Besset, Alain Combes, Adrien Robine, Marion Beuzelin, Julio Badie, Guillaume Chevrel, Jean Reignier, Julien Bohé, Elisabeth Coupez, Nicolas Chudeau, Saber Barbar, Christophe Vinsonneau, Jean-Marie Forel, Didier Thevenin, Eric Boulet, Karim Lakhal, Nadia Aissaoui, Steven Grange, Marc Leone, Guillaume Lacave, Saad Nseir, Florent Poirson, Julien Mayaux, Karim Asehnoune, Guillaume Geri, Kada Klouche, Guillaume Thiery, Laurent Argaud, Jean-Damien Ricard, Jean-Pierre Quenot, Didier Dreyfuss

**Affiliations:** 1 French National Institute of Health and Medical Research (INSERM), UMR_S1155, Remodeling and Repair of Renal Tissue, Hôpital Tenon, Sorbonne Université, F-75020 Paris, France; 2 AP-HP, Hôpital Avicenne, Service de Réanimation Médico-Chirurgicale, 125 Rue de Stalingrad, 93000 Bobigny, France; 3 Health Care Simulation Center, UFR SMBH, Université Paris 13, Sorbonne Paris Cité, Bobigny, France; 4 AP-HP, Hôpitaux Universitaires Pitié Salpêtrière-Charles Foix, Département Biostatistique Santé Publique et Information Médicale, Centre de Pharmacoépidémiologie (Cephepi), Sorbonne Université, INSERM, Institut Pierre Louis d’Epidémiologie et de Santé Publique, CIC-1421, F75013 Paris, France; 5 Réanimation polyvalente, CHR départementale La Roche Sur Yon, 85025 La Roche Sur Yon, France; 6 Réanimation polyvalente, CHR Metz-Thionville Hôpital de Mercy, 57085 Metz, France; 7 Réanimation polyvalente, CHG d’Avignon Henri Duffaut, 84902 Avignon, France; 8 Réanimation médicale, CHU d’Amiens Picardie, 80054 Amiens, France; 9 Réanimation, CH de Bretagne Sud, 56322 Lorient, France; 10 Réanimation, CHU Pointe-a-Pitre/Abymes, 97159 Pointe-a-Pitre, France; 11 Réanimation médicale, Hôpital Henri Mondor, 94010 Créteil, France; 12 Service de Réanimation Médico-Chirurgicale, AP-HP, Hôpital Louis Mourier, 178 rue des Renouillers, F-92700 Colombes, France; 13 Service de Réanimation Médicale, AP-HP, Hôpital Pitié Salpêtrière, 75013 Paris, France; 14 Réanimation Soins continus, CH de Bourg-en-Bresse – Fleyriat, 01012 Bourg-en-Bresse, France; 15 Réanimation polyvalente, CH de Dieppe, 76020 Dieppe, France; 16 Réanimation polyvalente, Hôpital Nord Franche-Comte CH Belfort, 90016 Belfort, France; 17 Réanimation polyvalente, CH Sud Francilien, 91106 Corbeil Essones, France; 18 Réanimation médicale, Hôtel Dieu, 44035 Nantes, France; 19 Anesthésie réanimation médicale et chirurgicale, CH Lyon Sud, 69495 Pierre Benite,, France; 20 Réanimation polyvalente, Hôpital G. Montpied, 63003 Clermont Ferrand, France; 21 Réanimation médico-chirurgicale, CH du Mans, 72037 Le Mans, France; 22 Réanimation, Hôpital Caremeau, 30029 Nimes, France; 23 Réanimation et USC, CH Bethune Beuvry – Bermont et Gauthier, 62408 Bethune, France; 24 Réanimation médicale, Hôpital Nord, 13015 Marseille, France; 25 Réanimation et USC, CH Dr Schaffner, 62307 Lens, France; 26 Réanimation et USC, GH Carnelle Portes de l’Oise, 95260 Beaumont sur Oise, France; 27 Anesthésie Réanimation, hôpital Nord laennec, 44093 Nantes, France; 28 Réanimation médicale, Hôpital Georges Pompidou, 75014 Paris, France; 29 Réanimation médicale, CHU Rouen, 76031 Rouen, France; 30 Anesthésie Réanimation, Hôpital Nord, 13015 Marseille, France; 31 Réanimation médico-chirurgicale, Hôpital André Mignot, 78000 Versailles, France; 32 Réanimation médicale, CHRU de Lille, Hôpital Roger Salengro, 59037 Lille, France; 33 Pneumologie et Réanimation médicale, Hôpital Pitié Salpêtrière, 75013 Paris, France; 34 Anesthésie-réanimation, Hôtel Dieu, 44035 Nantes, France; 35 Réanimation médico-chirurgicale, Hôpital Ambroise Paré, 92100 Boulogne-Billancourt, France; 36 Médecine Intensive Réanimation, Hôpital lapeyronnie, 34295 Montpellier, France; 37 Réanimation médicale, CHU Saint Etienne, 42270 Saint Priest en Jarez, France; 38 Réanimation médicale, Hôpital Edouard Herriot, 69437 Lyon, France; 39 Univ Paris Diderot, Sorbonne Paris Cité, IAME, UMRS 1137, F-75018 Paris, France; 40 INSERM, IAME, U1137, F-75018 Paris, France; 41 Department of Intensive Care, François Mitterrand University Hospital, Dijon, France; 42 Lipness Team, INSERM Research Center LNC-UMR1231 and LabExLipSTIC, University of Burgundy, Dijon, France; 43 INSERM CIC 1432, Clinical Epidemiology, University of Burgundy, Dijon, France; 44 Sorbonne Paris-Cité, Paris, France; 45 Present address: Intensive Care Unit, Hôpital Louis Mourier, 178 rue des Renouillers, 92110 Colombes, France

**Keywords:** Acute kidney injury, Critical care, Renal replacement therapy, Treatment outcome

## Abstract

**Background:**

The *Artificial Kidney Initiation in Kidney Injury* (AKIKI) trial showed that a delayed renal replacement therapy (RRT) strategy for severe acute kidney injury (AKI) in critically ill patients was safe and associated with major reduction in RRT initiation compared with an early strategy. The five criteria which mandated RRT initiation in the delayed arm were: severe hyperkalemia, severe acidosis, acute pulmonary edema due to fluid overload resulting in severe hypoxemia, serum urea concentration > 40 mmol/l and oliguria/anuria > 72 h. However, duration of anuria/oliguria and level of blood urea are still criteria open to debate. The objective of the study is to compare the delayed strategy used in AKIKI (now termed “standard”) with another in which RRT is further delayed for a longer period (termed “delayed strategy”).

**Methods/design:**

This is a prospective, multicenter, open-label, two-arm randomized trial. The study is composed of two stages (observational and randomization stages). At any time, the occurrence of a potentially severe condition (severe hyperkalemia, severe metabolic or mixed acidosis, acute pulmonary edema due to fluid overload resulting in severe hypoxemia) suggests immediate RRT initiation.

Patients receiving (or who have received) intravenously administered catecholamines and/or invasive mechanical ventilation and presenting with AKI stage 3 of the KDIGO classification and with no potentially severe condition are included in the observational stage. Patients presenting a serum urea concentration > 40 mmol/l and/or an oliguria/anuria for more than 72 h are randomly allocated to a standard (RRT is initiated within 12 h) or a delayed RRT strategy (RRT is initiated only if an above-mentioned potentially severe condition occurs or if the serum urea concentration reaches 50 mmol/l).

The primary outcome will be the number of RRT-free days at day 28.

One interim analysis is planned. It is expected to include 810 patients in the observational stage and to randomize 270 subjects.

**Discussion:**

The AKIKI2 study should improve the knowledge of RRT initiation criteria in critically ill patients. The potential reduction in RRT use allowed by a delayed RRT strategy might be associated with less invasive care and decreased costs. Enrollment is ongoing. Inclusions are expected to be completed by November 2019.

**Trial registration:**

ClinicalTrials.gov, ID: NCT03396757. Registered on 11 January 2018.

## Background

Acute kidney injury (AKI) is frequent among intensive care unit (ICU) patients and is associated with high morbidity and mortality [1]. Renal replacement therapy (RRT) is the cornerstone of severe AKI management [2]. The timing of RRT is one of the most debated issues in critical care medicine [3]. In the first, large-scale, single-center randomized controlled trial (RCT), 208 patients with community-acquired AKI were randomized to an early or “delayed” strategy for RRT initiation. Hospital mortality did not significantly differ between groups (12.2% vs 20.5% for the delayed strategy and early strategy, respectively, *p* = 0.2) but 17% of patients in the delayed group did not finally receive RRT [4]. More recently, the ELAIN trial (including 231 postsurgical patients) showed increased mortality in the delayed RRT group (*p* = 0.03). These results were potentially explained by the fact that a majority (almost 75%) of patients had fluid overload or worsening pulmonary edema at baseline while most authorities consider that severe pulmonary edema is an absolute indication for emergent RRT [5].

The *Artificial Kidney Initiation in Kidney Injury* (AKIKI) trial [6] published in 2016 showed that delayed RRT initiation did not result in lower mortality as compared to an early initiation strategy [7]. An important finding was that nearly 50% of patients escaped RRT in the delayed strategy. Additionally, renal function recovery occurred earlier and catheter-related infections were less frequent in the delayed group. The *Initiation of Dialysis EArly Versus deLayed in Intensive Care Unit* (IDEAL-ICU) trial [8] confirmed the results of AKIKI in a septic-shock patient population [9]. Findings of these two large, multicenter RCTs strongly suggest considering the delayed RRT initiation strategy as the standard of care [10].

The five criteria which mandated RRT initiation in the delayed arm of AKIKI (severe hyperkalemia, severe metabolic or mixed acidosis, acute pulmonary edema due to fluid overload resulting in severe hypoxemia, serum urea concentration > 40 mmol/l and oliguria/anuria for more than 72 h after randomization) had different degrees of severity. The first three criteria represented potentially life-threatening situations but the majority of RRT indications in the delayed group of AKIKI stemmed from the two others (duration of anuria and level of serum urea). However, neither the duration of anuria/oliguria, nor the level of blood urea have ever been demonstrated as robust indications for RRT.

The objective of the study is to compare the delayed strategy used in AKIKI (now termed “standard”) with another in which RRT is further delayed (in the absence of a life-threatening complication as defined above) for a longer period (this group will be termed delayed strategy) (ClinicalTrials.gov, ID: NCT03396757).

## Methods/design

### Design and settings

**Fig. 1:**
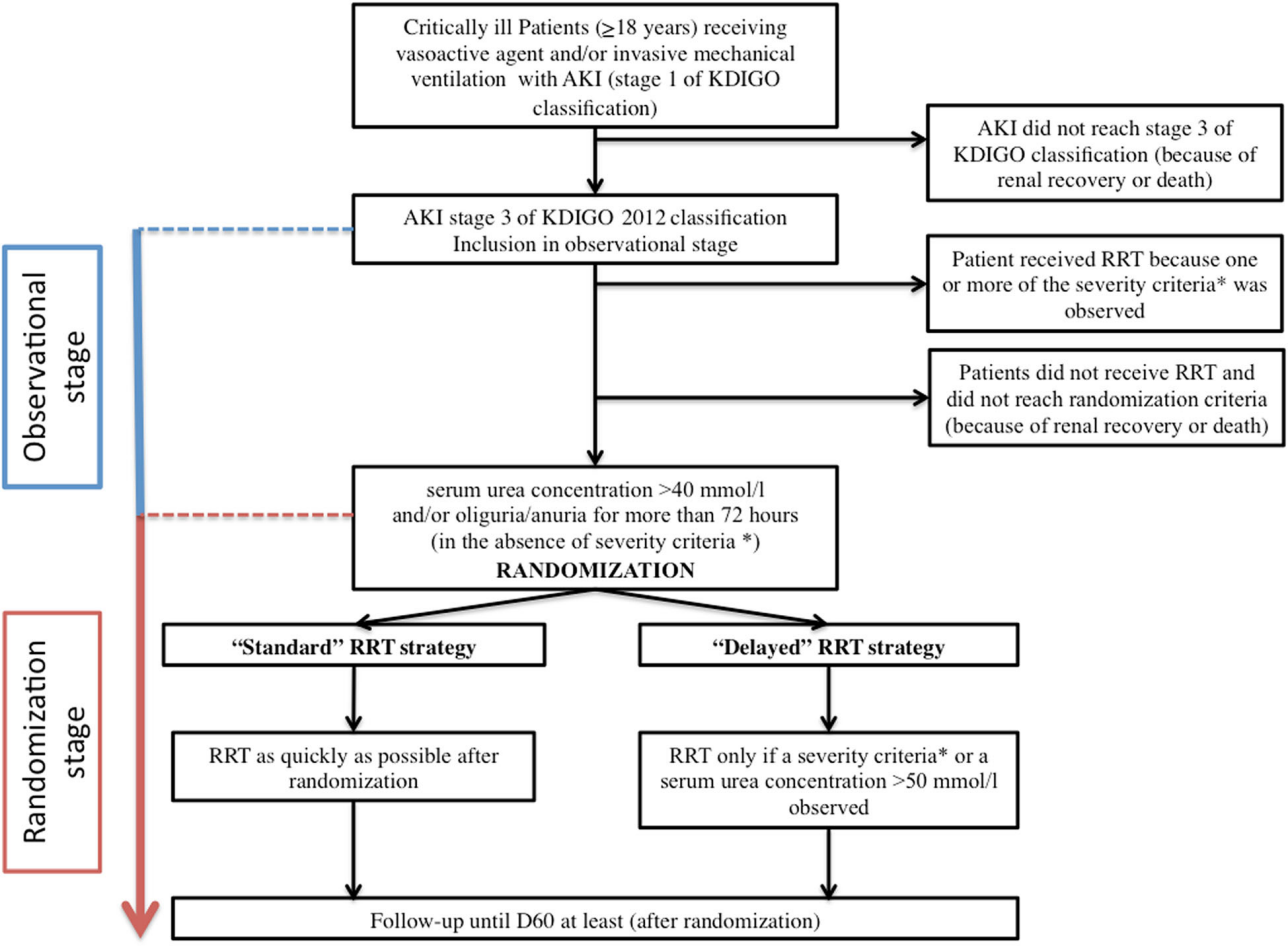
Study design. The AKIKI 2 trial is composed by 2 stages (observational and randomization stages). *Severity criteria which make considering renal replacement therapy (RRT) initiation (see Table 1): serum potassium concentration > 6 mmol/l, serum potassium concentration > 5.5 mmol/l persisting despite medical treatment, arterial blood pH < 7.15 in a context of pure metabolic acidosis (PaCO_2_ < 35 mmHg) or in a context of mixed acidosis with a PaCO_2_ > 50 mmHg without the possibility of increasing alveolar ventilation, acute pulmonary edema due to fluid overload despite diuretic therapy leading to severe hypoxemia requiring oxygen flow rate > 5 l/min to maintain SpO_2_ > 95% or FiO_2_ > 50% under invasive or non-invasive mechanical ventilation

**Fig. 2:**
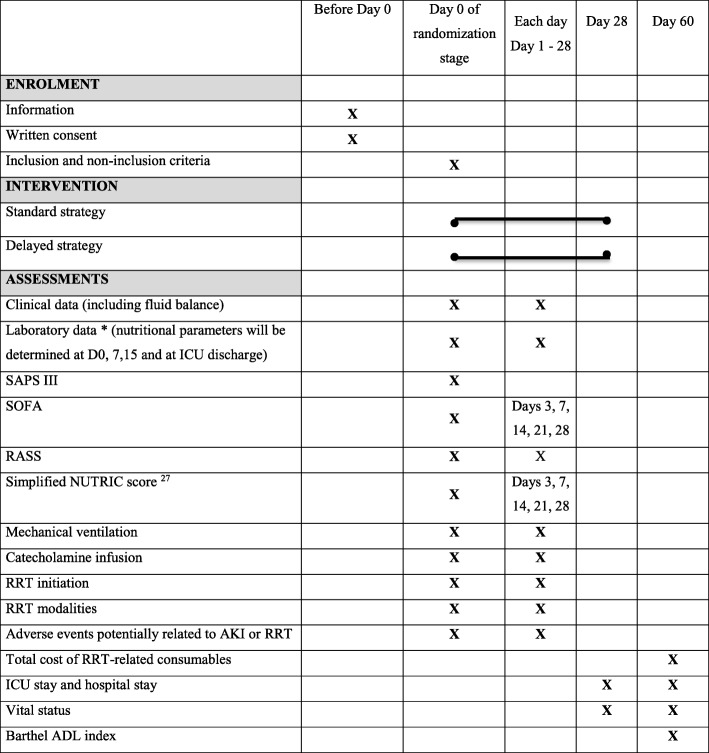
Chronology of the research (Standard Protocol Items: Recommendations for Interventional Trials (SPIRIT) Figure)

The AKIKI 2 is a prospective, multicenter, open-label, two-arm randomized trial. The study is composed by two stages (observational and randomization stages) (see Fig. 1). At any time during these two stages, the occurrence of a potentially severe condition defined in the Table 1 suggests the need for immediate RRT initiation unless a medical treatment can very rapidly resolve the situation. The trial accords with the Standard Protocol Items: Recommendations for Interventional Trial (SPIRIT) guidelines (see Fig. 2 Additional file 1.).

**Table 1:** Criteria that make considering renal replacement therapy (RRT) initiation at any time during the 2 stages of the study

Serum potassium concentration > 6 mmol/l	
Serum potassium concentration > 5.5 mmol/l persisting despite medical treatment	
Arterial blood pH < 7.15 in a context of pure metabolic acidosis (PaCO_2_ < 35 mmHg) or in a context of mixed acidosis with a PaCO_2_ > 50 mmHg without possibility of increasing alveolar ventilation	
Acute pulmonary edema due to fluid overload despite diuretic therapy leading to severe hypoxemia requiring oxygen flow rate > 5 l/min to maintain SpO_2_ > 95% or FiO_2_ > 50% under invasive or non- invasive mechanical ventilation

### Ethical aspects

The study protocol and information forms were approved by the competent French legal authority (*Comité de Protection des Personnes Sud Est V, 7 February 2018*).

#### Observational stage

For this stage, patients are informed both verbally and with a written document about the observational stage by the principal investigator or a physician representing the investigator, before the patient is enrolled on the study. Only an oral consent will be required for this stage in accordance with French law. If the patient is unable to receive appropriate information, a decision is made by a substitute decision-maker; in descending order of priority, a legal representative, a family member or a close relative. Patients who are eligible but incapable of receiving information and for whom a substitute decision-maker is not present may be included through a process of deferred information. Substitute decision-makers are informed as soon as possible and patients are informed about participation as soon as their clinical status allows. Patients or surrogates are eventually informed about the trial and their right to refuse participation.

#### Randomization stage

Written informed consent is obtained by the principal investigator or a physician representing the investigator, before the person is enrolled on the study. If the patient is unable to receive appropriate information, a decision is made by a substitute decision-maker. Patients who are eligible but incapable of receiving information and for whom a substitute decision-maker is not present may be included through a process of deferred consent. The substitute decision-maker will be informed as soon as possible and patients will be informed about participation as soon as their clinical status allows. Their consent for continuing participation is sought.

### Participating ICUs

Thirty-six French ICUs will participate to the study. All study sites have experienced medical and paramedical teams in the field of RRT as most of them participated in previous studies [7, 9]. The choice of RRT modality is left to the team’s discretion depending on its practices. In each study site, RRT prescription and monitoring are standardized according to the French national guidelines [11].

### Study population

Eligible patients are adults (aged > 18 years) with AKI (stage 1 of the Kidney Disease: Improving Global Outcomes (KDIGO) classification) compatible with the diagnosis of acute tubular necrosis in a context of ischemic or toxic aggression and receiving (or who have received for the present episode) invasive mechanical ventilation and/or catecholamine infusion.

To be included in the observational stage, patients must meet at least one of the three following criteria: serum creatinine concentration > 354 μmol/l or greater than three times the baseline creatinine level, anuria (urine output < 100 ml) for more than 12 h, oliguria (urine output < 0.3 ml/kg/h or < 500 ml/day) for more than 24 h. These criteria represent stage 3 of the KDIGO classification.

To be randomized (randomization stage), patients must meet at least one the supplemental criteria: oliguria/anuria (urine output < 0.3 ml/kg/h or < 500 ml/day) for more than 72 h or serum urea concentration of between 40 and 50 mmol/l.

Patients presenting one of the emergent indications for immediate RRT (Table 1) are not included. Other non-inclusion criteria are: serum urea level > 50 mmol/l; severe chronic renal failure (creatinine clearance < 30 ml/min); patients with the inclusion criteria already present for more than 24 h (to avoid delayed inclusions); AKI caused by urinary tract obstruction or renal vessel obstruction or tumor-lysis syndrome or thrombotic microangiopathy or acute glomerulopathy; poisoning by a dialyzable agent; Child C liver cirrhosis; cardiac arrest without awakening; moribund state (patient likely to die within 24 h); patient having already received RRT for the current episode of AKI; renal transplant; treatment limitation (withholding or withdrawal); previous inclusion in this study; subject deprived of freedom, subject under a legal protective measure; and pregnant or breastfeeding woman. These non-inclusion criteria should not be present at the time of inclusion in the observational stage of the study, and should not have appeared secondarily at the time of randomization.

### Observational stage

All patients receiving (or who have received for the present episode) intravenously administered catecholamines and/or invasive mechanical ventilation and presenting with AKI classification stage 3 of the KDIGO classification and with no potentially life-threatening condition mandating immediate RRT start as described in Table 1 will be included in the observational stage. Clinical and metabolic conditions will be closely monitored and RRT will be mandatory if one or more of the potentially life-threatening conditions (Table 1) occurs.

### Randomization stage

Patients presenting one or both of following criteria: a serum urea concentration > 40 mmol/l but < 50 mmol/l and/or an oliguria/anuria (oliguria = urine output < 0.3 ml/kg/h or < 500 ml/day; anuria = urine output< 100 ml/day) for more than 72 h without any above-mentioned (Table 1) life- threatening condition are randomly allocated to one of the two study treatment arms, termed “standard” strategy and “delayed” RRT strategy. Randomization and concealment are achieved using a centralized, secure, computer-generated, interactive, web-response system accessible from each study center. Randomizations are balanced by blocks of variable and undisclosed size and stratified on the center. Before randomization, the presence of the inclusion criteria and the absence of the non-inclusion criteria are verified.

### Study interventions (for randomization stage)

*Standard strategy*: RRT is initiated within 12 h after documentation of serum urea concentration > 40 mmol/l and/or an oliguria/anuria (urine output < 0.3 ml/kg/h or < 500 ml/day) for more than 72 h. The timing of initiation is recorded and RRT will continue until criteria for cessation are observed (see below).

*Delayed strategy*: RRT initiation is strongly suggested if one or more of the above-mentioned potentially severe situations (Table 1) occurs or if the serum urea concentration reaches 50 mmol/l. Patients do not receive RRT whatever the duration of anuria/oliguria if any of the above-mentioned indications for RRT is not present. The decision to initiate RRT in the delayed strategy arm of the trial will have to be approved by the attending physician(s) involved in the patient’s care.

### Renal replacement therapy delivery and cessation

The choice of RRT modality (intermittent or continuous technique) is left to the study site discretion. Several RRT modalities can be used in the same patient, according to the attending physician’s indication. The duration of, and interval between, sessions, and device settings, as well as the modality of anticoagulation, are left to the investigator’s discretion.

In case of RRT initiation in a context of high serum urea concentration (> 40 mmol/l), prevention of dialysis disequilibrium syndrome will be recommended (even if it is usually recommended for chronic hyper-uremia). The management will be left to the clinician’s discretion and will include one or several of the following measures: slow, gentle initial hemodialysis (dialysis time < 2 h and low blood-flow rate); increasing dialysate sodium levels; administration of an osmotically active substance by using a high-glucose-concentration dialysate or administering hypertonic glucose via the venous line of the dialyzer during dialysis.

All study centers have extensive experience in both AKI management and RRT techniques.

Renal replacement therapy discontinuation is contemplated when spontaneous diuresis > 500 ml/24 h, and highly recommended if diuresis is > 1000 ml/24 h without diuretic administration or > 2000 ml/24 h, in patients receiving diuretics.

Renal replacement therapy cessation will be mandatory if diuresis (as defined above) is present and serum creatinine level decreases spontaneously.

If an improvement of renal function is insufficient to achieve a spontaneous decrease in creatinine level and/or if diuresis becomes lower than 1000 ml/24 h without diuretics (or lower than 2000 ml/24 h under diuretics), RRT is resumed.

Renal function recovery is defined in three different ways:

- Adequate diuresis (> 1000 ml per 24 h in the absence of diuretic therapy or > 2000 ml/ per 24 h with diuretic therapy) and no RRT initiation or resumption for at least 7 days
- Spontaneous decrease of serum creatinine value and no RRT initiation or resumption for at least 7 days
- Absence of any of AKI KDIGO stages 1–3 (we will also perform a subgroup analysis with the absence of AKI stages 2–3) [12]

### Authorized treatments (whatever the randomization arm)

#### Pharmacological prevention of gastro-intestinal bleeding

Given the risk factors for digestive bleeding in this population (mechanical ventilation and/or need for catecholamine infusion in patients with severe AKI), and in the absence of consensus on this issue in available guidelines, the protocol will recommend the use of a proton-pump inhibitor.

#### Use of diuretics

Loop diuretics should only be used for the treatment of obvious sodium and fluid overload in patients whose diuresis is < 500 ml/24 h.

#### Hyperkalemia treatment

Enteral administration of sodium polystyrene sulfonate and infusion of sodium bicarbonate or glucose-insulin, or beta-2-mimetic aerosols to treat hyperkalemia are left to the clinician discretion and closely monitored. In case of associated acidosis, minute ventilation delivered by the respirator is increased, when feasible.

#### Nutrition

Based on international guidelines [13], a modified NUTRIC score [14] will be assessed. All study sites adhere to guidelines on nutrition in accordance with national and international guidelines [13, 15–17]. During the ICU stay, nutritional intake is closely monitored and serum albumin, transthyretin and C-reactive protein (CRP) are assessed at days 0, 7 and 15 and at ICU discharge.

### Data collection and follow-up

At D0, demographic data and medical history, including the current clinical history with the reason for ICU admission, Simplified Acute Physiology Score (SAPS) III severity score [18] and Sequential Organ Failure Assessment score (SOFA) score [19] are collected. Potential exposure to nephrotoxic agents (e.g., aminoglycosides or contrast agents) is documented. Treatments including mechanical ventilation (and its settings), fluid therapy, catecholamine and anticoagulant administration are recorded. The Richmond Agitation-sedation Scale (RASS) [20] is assessed. Laboratory tests include serum (and urine if diuresis is present) electrolyte-level determination, serum glucose level, urea and creatinine concentration, and arterial blood gas determination. Samples of plasma and urine are collected to create a biobank. Baseline serum creatinine concentrations are determined by either results of a measurement in the 12 months preceding the ICU stay or estimation using the Modification of Diet in Renal Disease (MDRD) study equation assuming that baseline eGFR is 75 ml/min per 1.73 m^2^ [21]. *Special case*: if the serum creatinine concentration was measured more than 12 months before admission, the baseline level will be considered as the highest of the two estimations (former serum concentration or that computed by the MDRD formula).

From D1 to D28, the same biological data as those collected at inclusion are recorded according to clinical indications for routine care until ICU discharge or day (D) 28 in the ICU, or death in the ICU. Search for infection (including catheter-related infection) or complications related to RRT or AKI are carried out according to routine care procedures. The SOFA score is also calculated at D3, D7, D14, D21 and D28. The RASS is assessed each day during ICU stay.

At D60, vital status (alive or dead), duration of hospital and ICU stay, Barthel ADL Index [22] are recorded.

### Organization of the trial

#### Funding/support

The AKIKI2 trial is promoted by the Assistance Publique – Hôpitaux de Paris and supported by a grant from the French Ministry of Health (Programme Hospitalier de Recherche Clinique 2016; AOM16278).

#### Coordination and implementation of the trial

Each medical and paramedical team of the 36 participating ICUs were trained to the protocol and data collection in the electronic Case Record Form (eCRF) during formal meetings prior to the start of screening and inclusion. The eCRF is developed with CleanWEB™, a centralized, secure, interactive, web-response system accessible from each study center, provided and managed by Telemedicine technologies.

Local physicians and clinical research assistants in each participating ICU are responsible for the daily screening and inclusion of patients, compliance with the protocol, availability of data requested for the trial and completion of the eCRF. In accordance with French law, the eCRF and database were validated by the appropriate committees (CCTIRS: Comité Consultatif sur le Traitement de l’Information en matière de Recherche dans le domaine de la Santé; CNIL: Commission Nationale de l’Informatique et des Libertés).

#### Interim analysis

An interim analysis of safety by an independent Data Safety and Monitoring Board (DSMB) is planned after randomization of 135 subjects. The DSMB will be blinded to the allocation of groups. This DSMB consists of one intensivist with special competency in methodology, one nephrologist and one intensivist. Data are blindly analyzed but unblinding is possible upon request of the DSMB. The DSMB will examine D-60 mortality and the rate of complications. An extraordinary meeting may be requested by the principal investigator or the methodologist in case of unexpected events that might affect continuation of the protocol.

### Blinding

Given the nature of the interventions, physicians, nurses and patients cannot be blinded for the randomized interventions. The analysis will be blinded to allocation of groups.

### Study outcomes

#### Primary endpoint

The primary outcome will be the number of RRT-free days at D28 (after randomization). One point will be given for each calendar day during the measurement period (i.e. from the first day of randomization to D28) that a patient was both alive and free of RRT, assuming that the patient survives and remains free of RRT for at least three consecutive calendar days after RRT weaning, whatever the vital status at D28. Zero value will be given for patients with RRT initiated on the first day of randomization who died before RRT weaning or who remained under RRT until D28.

#### Secondary endpoints

Secondary endpoints related to the randomization stage will be D-60 mortality; the percentage of patients receiving RRT at least once in the delayed RRT strategy arm; number of RRT sessions (until D28 after randomization) (analyzing alive or dead patients separately); time between randomization and RRT initiation; time to RRT weaning and to renal function recovery as defined above; number of dialysis catheter-free days between D0 and D28; rate of catheter-related (both dialysis and non-dialysis catheters) bloodstream infection; Barthel ADL Index 20 score at D60; percentage of patients on withholding and withdrawal of life support therapies (W-WLST); hydration status (weight, edema scale, fluid balance); nutritional status evaluated by the amount of calories and protein administered and by serum albumin, transthyretin and CRP concentration changes; number of hemorrhages requiring red blood cell transfusion or surgical procedure; rate of adverse events potentially related to AKI or RRT: (a) thrombocytopenia (< 100,000 platelets/mm^3^), (b) thrombosis of a large venous axis diagnosed by Doppler ultrasonography or computed tomography (CT) scan, (c) hypokalemia (defined as serum potassium concentration < 3 mmol/l), (d) hypophosphatemia (defined as a serum phosphate concentration < 0.6 mmol/l), (e) hyperkalemia (> 6.5 mmol/l), (f) hyponatremia (< 125 mmol/l), (g) hypernatremia (> 150 mmol/l), (h) cardiac rhythm disorders (ventricular tachycardia, ventricular fibrillation, *torsade de pointes* or new episode of atrial fibrillation requiring medical treatment or external electric counter shock), (i) pneumothorax, (j) hemothorax, (k) air embolism, (l) arteriovenous fistula, (m) pericarditis, (n) unexpected cardiac arrest and (o) hypothermia (< 34 °C).

Secondary endpoints related to the whole study population (observational and randomization stage) will be the duration of ICU and hospital stay (limited to D60); death in ICU, at D28, D60 and in-hospital; ventilator-free days at day 28; RRT indications; RRT modalities (continuous renal replacement therapy (CRRT), intermittent hemodialysis (IHD), other); duration of RRT; time to renal function recovery.

### Statistical methods

#### Sample size calculation

For the randomization stage, the hypothesis used for sample size calculations is derived from the results of the *delayed* arm of the AKIKI trial [7]: the mean number of RRT-free days at day 28 was 17 ± 11.4 days. This arm is now the *standard arm* of the current study. We made the assumption that further delaying RRT, a strategy now called *delayed strategy* (by contrast with the above-defined standard strategy) will increase the number of RRT-free days to 21 days (an increase of 4 days, i.e., approximately 25%). Considering a drop-out rate of approximately 5%, total sample size required is 270 (135 in each group) to detect this difference with 80% power (alpha = 5%, bilateral formulation). This is the required (and planned) sample size of the randomized study (the second stage). To compute the number of patients to be enrolled in the observation stage in order to achieve 270 of them reaching the randomization phase, we made the following calculations. Three hundred and eight patients were included in the *delayed* arm of the AKIKI trial [7] which corresponds now to the standard arm in this study. Among this population, 151 patients died or recovered renal function before the attainment of any RRT criteria, and 157 required RRT. Among them, 61 patients required RRT because of life-threatening situations (severe hyperkalemia, severe metabolic or mixed acidosis, acute pulmonary edema due to fluid overload resulting in severe hypoxemia). Thus, approximately one third (96/308) of patients included in the *delayed* arm of the AKIKI trial would have been eligible for the randomization stage of the AKIKI2 study, and this is the best possible approximation. Therefore, to be able to randomize 270 subjects in the second stage, 810 patients may be required in the observational stage. In all cases, enrollment will be stopped after the randomization of 270 subjects, even if this leads to increasing or decreasing the number of patients included in the first stage.

#### Interim analysis

An interim analysis of safety by an independent DSMB is planned after the follow-up completion of the first 135 patients included in the randomized stage. The D-60 survival rates and rates of complications will be compared between the two randomization groups using the chi^2^ or Fisher’s test, as appropriate. There are no predefined criteria for stopping the trial but the independent DSMB will be free to suggest early termination.

The survival rate and complications are not the primary outcome of this study, so no specific adaptation of the sample size is necessary to maintain an overall type-I error rate of 5%.

#### Analysis of the primary endpoint

The primary endpoint is the number of RRT-free days at D28 assessed among the subjects included in the randomization stage. The normality of the distribution will be checked using a Q-Q plot and a Shapiro-Wilk test because this type of variable is commonly skewed. If appropriate, RRT-free days will be described using median and interquartile range (25th percentile–75th percentile), and compared between the two groups using a non-parametric Wilcoxon signed-rank test. The mean, the standard deviation, and the Student’s *t* test will be used if the normality assumption is not rejected.

#### Analysis of secondary endpoints (randomization stage)

Categorical endpoints (rate of adverse events and rate of RRT) will be compared using the chi^2^ or Fisher’s test, as appropriate. Continuous endpoints (hydration and nutrition status, serum albumin, transthyretin and CRP concentrations, number of hemorrhages, number of RRT sessions, number of dialysis catheter-free days, number of ventilation-free days) will be compared using the Student’s *t* or Wilcoxon test, as appropriate. Time-to-event endpoints (overall survival, time to RRT initiation, time to RRT weaning) will be compared using the log-rank test.

These primary and secondary endpoints will be assessed among the subjects included in the randomization stage, and analyzed in the intention-to-treat (ITT) population.

#### Analysis of secondary endpoints (whole population)

A descriptive analysis of patients’ outcomes and of RRT indications and modalities in the whole study population (i.e., all patients included in the observational stage, whether they are eventually included in the randomized stage or not) will be performed.

Prognostic factors of death and predictive factors of RRT initiation and renal function recovery will be assessed using univariate and multivariate Cox regression.

All analyses will be performed at the bilateral alpha risk of 5%, using R software (R Foundation for Statistical Computing, Vienna, Austria.) version 3.2.3 or later, or SAS version 9.2 or later.

## Discussion

Investigations on RRT indications in the context of AKI in critically ill patients are considered a priority in clinical research [3]. Recent large, multicenter RCTs [7, 9] have shown that in the absence of life-threatening conditions (severe hyperkalemia, severe metabolic acidosis or pulmonary edema due to fluid overload in context of anuria) RRT could be safely delayed and that this dramatically reduces the number of patients receiving RRT. STARRT-AKI, an ongoing multicenter international trial (NCT02568722) will be the largest study comparing immediate and delayed strategy for initiating RRT. However, the term “delayed” is imprecise as it was 1 day in the pilot study and nearly 3 days in AKIKI. AKIKI2 aims at further extending this delay. Indeed, in previously published RCTs a significant number of patients included in a delayed arm finally received RRT after 2 or 3 days despite the absence of any life-threatening condition. Initiation of RRT was (by protocol) motivated by the duration of anuria (> 2 or 3 days) or the level of serum urea (> 40 mmol/l). The pertinence of these indications has never been rigorously evaluated. It therefore seems necessary to test the hypothesis that, in the absence of life-threatening conditions, RRT might be safely delayed beyond 3 days even if renal function has not recovered. This will potentially further reduce needless RRT initiation in critically ill patients.

The aim of reducing RRT exposition is supported by recent interesting findings. Beyond the evidence that RRT is associated with catheter-related complications (infections, hemorrhages, pneumothorax, arterio-venous fistula) and intra-dialytic hypotension, it has been recently underlined that it could delay renal function recovery after an episode of AKI [23]. This has led to the emergence of a new concept: the artificial kidney-induced kidney injury concept [10]. Moreover, decreasing the use of needless RRT in ICU patients would allow for significant cost reduction.

## Trial status

Inclusions started in May 2018. Enrollment is ongoing. The interim analysis was conducted in June 2019, and the DSMB recommended to continue the study. The last version of the protocol is 3.0, 1 March 2019. Inclusions are expected to be completed in November 2019.

## Supplementary information

**Additional file 1.** Standard Protocol Items: Recommendations for Interventional Trials (SPIRIT) 2013 Checklist: recommended items to address in a clinical trial protocol and related documents*.

## Data Availability

Not applicable

## Abbreviations

AKI: Acute kidney injury
Barthel ADL Index: Barthel activities of daily living
CRP: C-reactive protein
DSMB: Data Safety and Monitoring Board
e-CRF: Electronic Case Record Form
ICU: Intensive care unit
MDRD: Modification of diet in renal disease
NUTRIC score: Nutrition risk in critically ill score
RASS: Richmond Agitation-sedation Scale
RCTs: Randomized controlled trials
RRT: Renal replacement therapy
SAPS III: Simplified Acute Physiology Score
SOFA score: Sequential Organ Failure Assessment score
W-WLST: Withholding and withdrawal of life support therapies
